# Supplementary figures and images for: The DNA helicase FANCJ (BRIP1) functions in double strand break repair processing, but not crossover formation during prophase I of meiosis in male mice
Source: PLoS Genet. 2024 Feb 20;20(2):e1011175. doi: 10.1371/journal.pgen.1011175 (PMC10906868; doi:10.1371/journal.pgen.1011175)

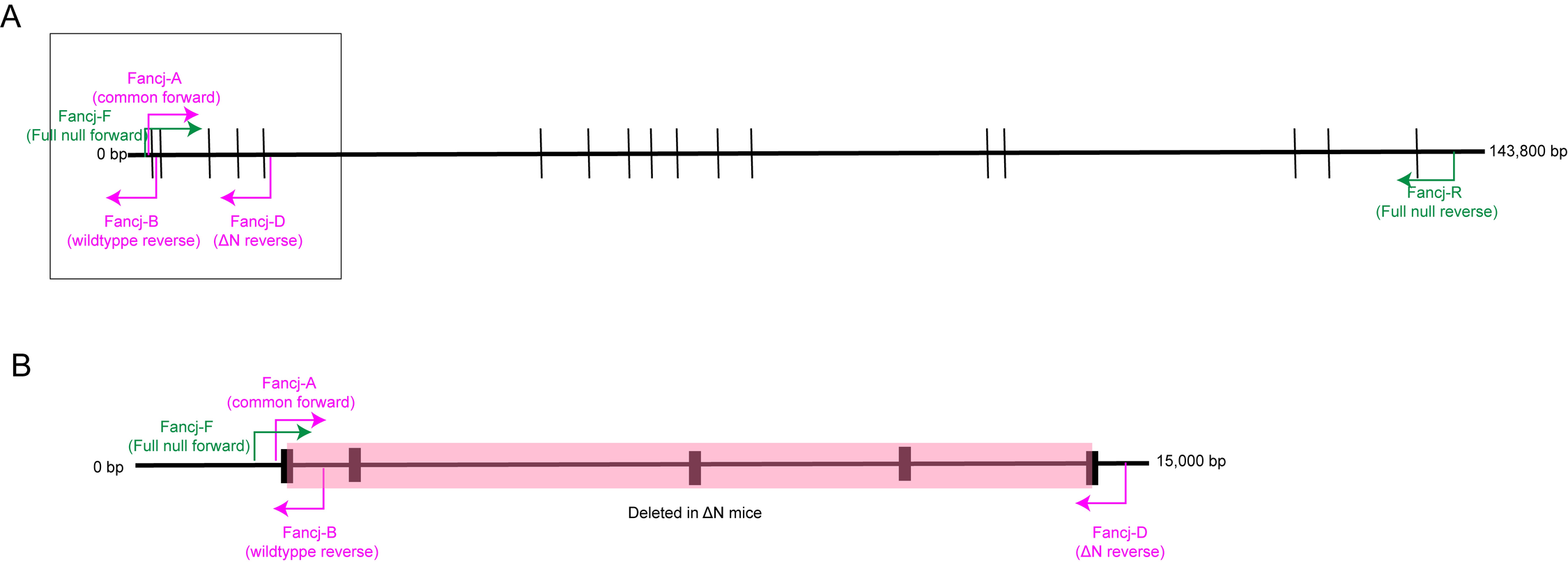

Supplement: S1 Fig — A) Diagram of the murine Fancj gene. Vertical black lines denote exons; pink arrows show binding sites of the primers used to genotype the ΔN allele while green arrows show binding sites of the primers used to genotype the full gene deletion of Fancj. B) A zoomed-in section (marked by the box in A) showing the binding sites of the Fancj-ΔN genotyping primers and the forward primer for Fancj full gene deletion genotyping. (TIF) [file pgen.1011175.s001.tif]

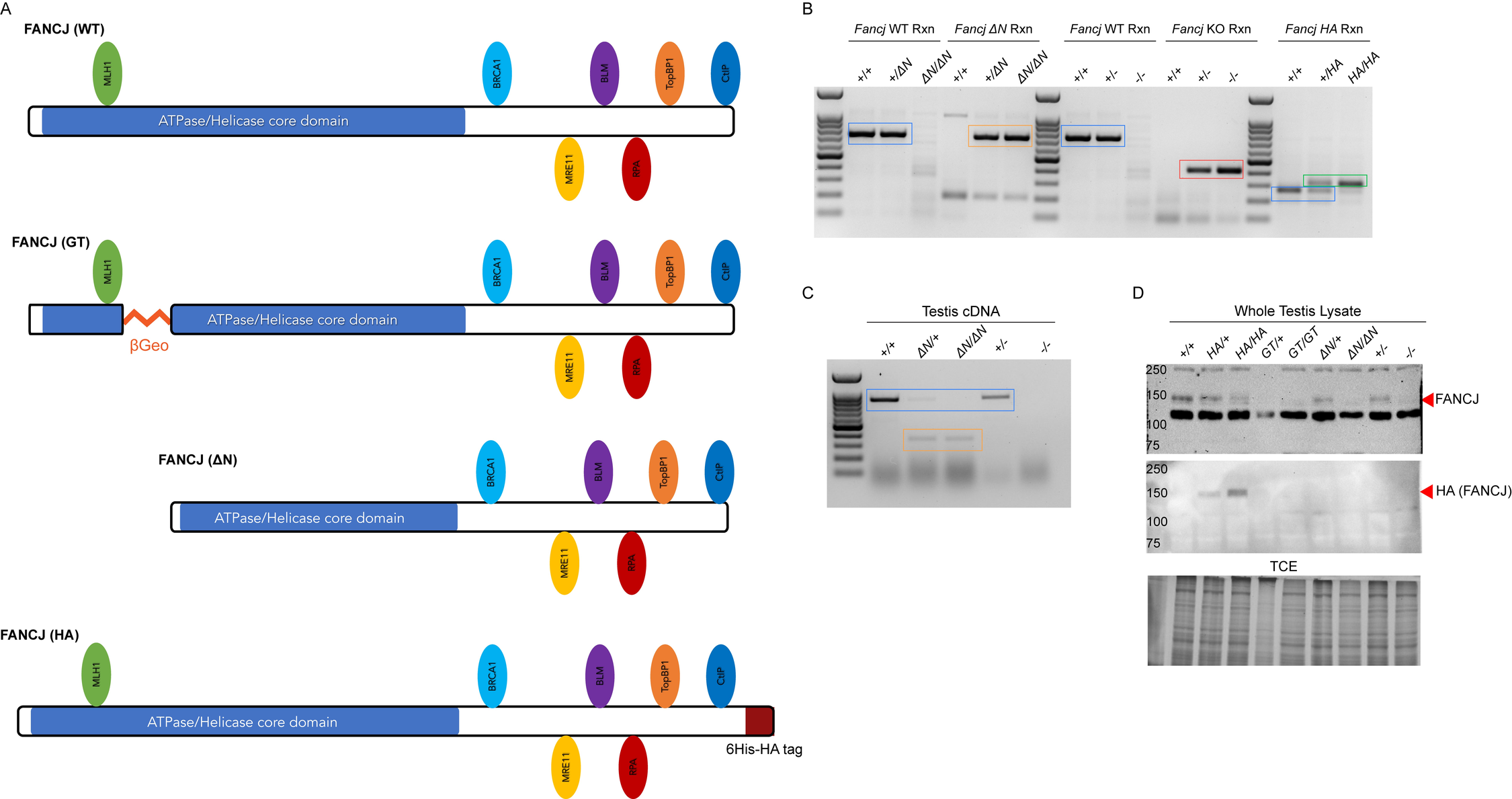

Supplement: S2 Fig — A) Diagram detailing the wildtype, gene-trap, ΔN, and epitope-tagged FANCJ proteins with their predicted protein interaction sites (The full deletion is excluded as it does not make a protein). The FANCJ-ΔN protein in missing the N-terminal region of the ATPase/Helicase core domain and the MLH1 interaction site, while the epitope-tagged protein has a 6xHis-HA dual tag at the C-terminus. B) PCR genotyping of FancjΔN, Fancj-, and FancJHA mutants. PCR product bands are boxed in colors pertaining to allele: Fancj+ (blue), FancjΔN (orange), Fancj- (red), and FancjHA (green). C) PCR of testis cDNA from Fancj+/+, Fancj+/ΔN, FancjΔN/ΔN, Fancj+/-, and Fancj-/- mice. PCR product bands are boxed in colors pertaining to allele coding sequence: Fancj+ (blue), FancjΔN (orange). As expected, no band was detected for Fancj-, indicting no transcript was made. D) Western blots using whole testis lysate from each Fancj genotype: Fancj+/+, FancjHA/+, FancjHA/HA, FancjGT/+, FancjGT/GT, FancjΔN/+, FancjΔN/ΔN, Fancj+/-, and Fancj-/-. Lysates were run on an 8% bis-acrylamide, 0.5% TCE gel. Top depicts the membrane blotted with rabbit antibody to FANCJ; middle is the same membrane after being stripped and reblotted with mouse antibody to HA; and bottom is the TCE loading control. Red arrows indicate FANCJ and HA bands (expected size: 131 kDa but detected at 150 kDa). (TIF) [file pgen.1011175.s002.tif]

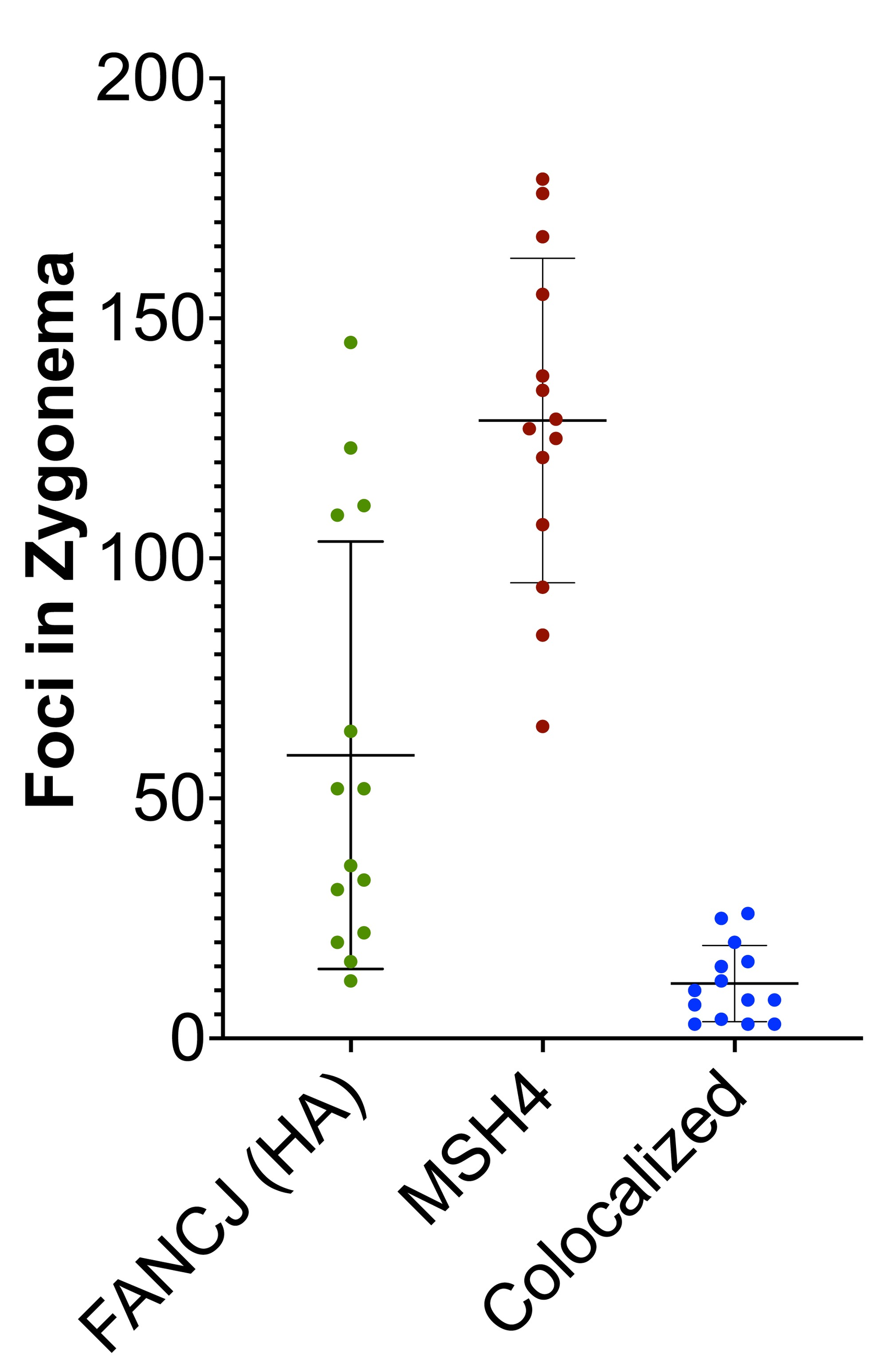

Supplement: S3 Fig — Quantification of colocalization between FANCJ-HA and MSH4 in zygonema (n = 3 males, 14 total cells analyzed). Bars represent mean ± SD. (TIF) [file pgen.1011175.s003.tif]

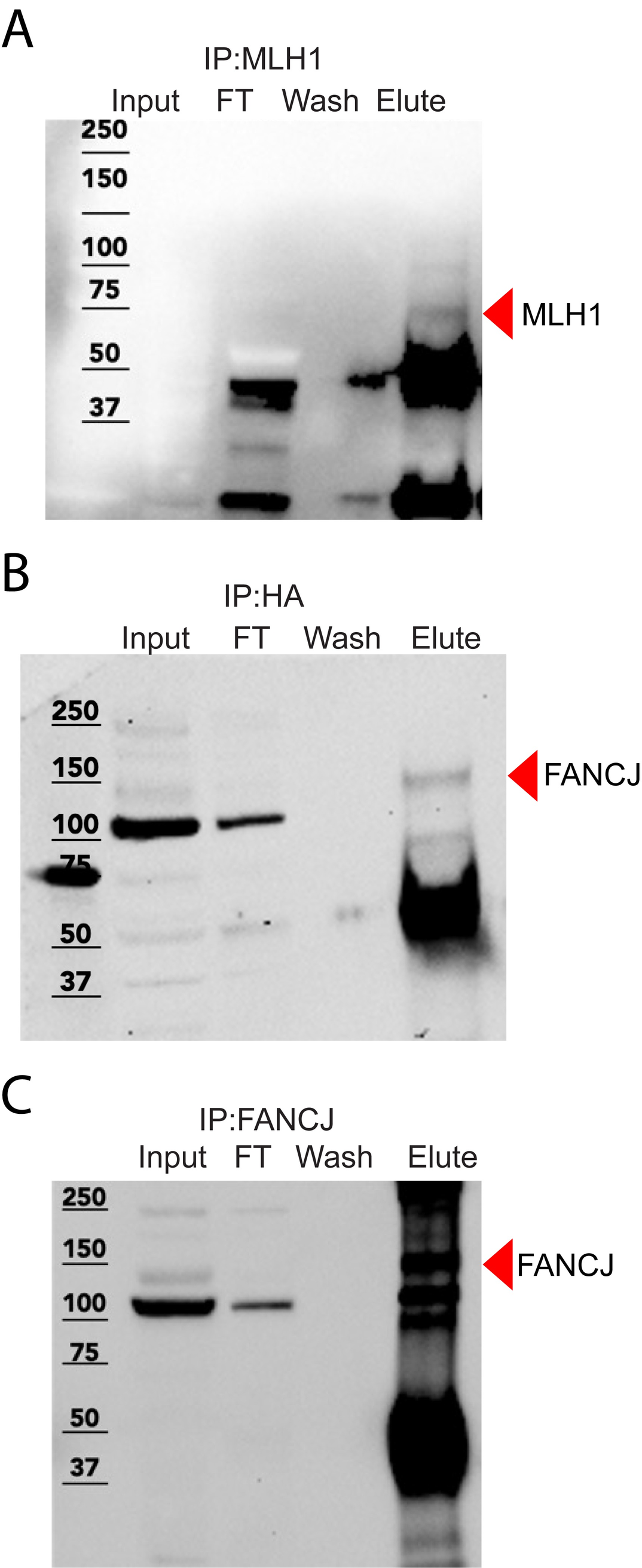

Supplement: S4 Fig — Reciprocal IP-WBs from those shown in Fig 5A) Western blot of MLH1-IP using whole testis lysate input. Sample was run on an 8% SDS-PAGE gel, and membrane blotted with antibody against MLH1. Black arrow points to band corresponding to MLH1 in the elution. B) Western blot of HA-IP using FancjHA/HA whole testis lysate input. Sample was run on an 8% SDS-PAGE gel, and membrane blotted with antibody against FANCJ. Black arrow highlighting FANCJ bands in elution. C) Western blot of FANCJ-IP using whole testis lysate input. Sample was run on an 8% bis-acrylamide gel, and membrane blotted with antibody against FANCJ. Black arrow highlighting FANCJ bands. Two biological replicates were performed for MLH1-IPs and three biological replicates were performed for FANCJ-IP and HA-IPs. (TIF) [file pgen.1011175.s004.tif]

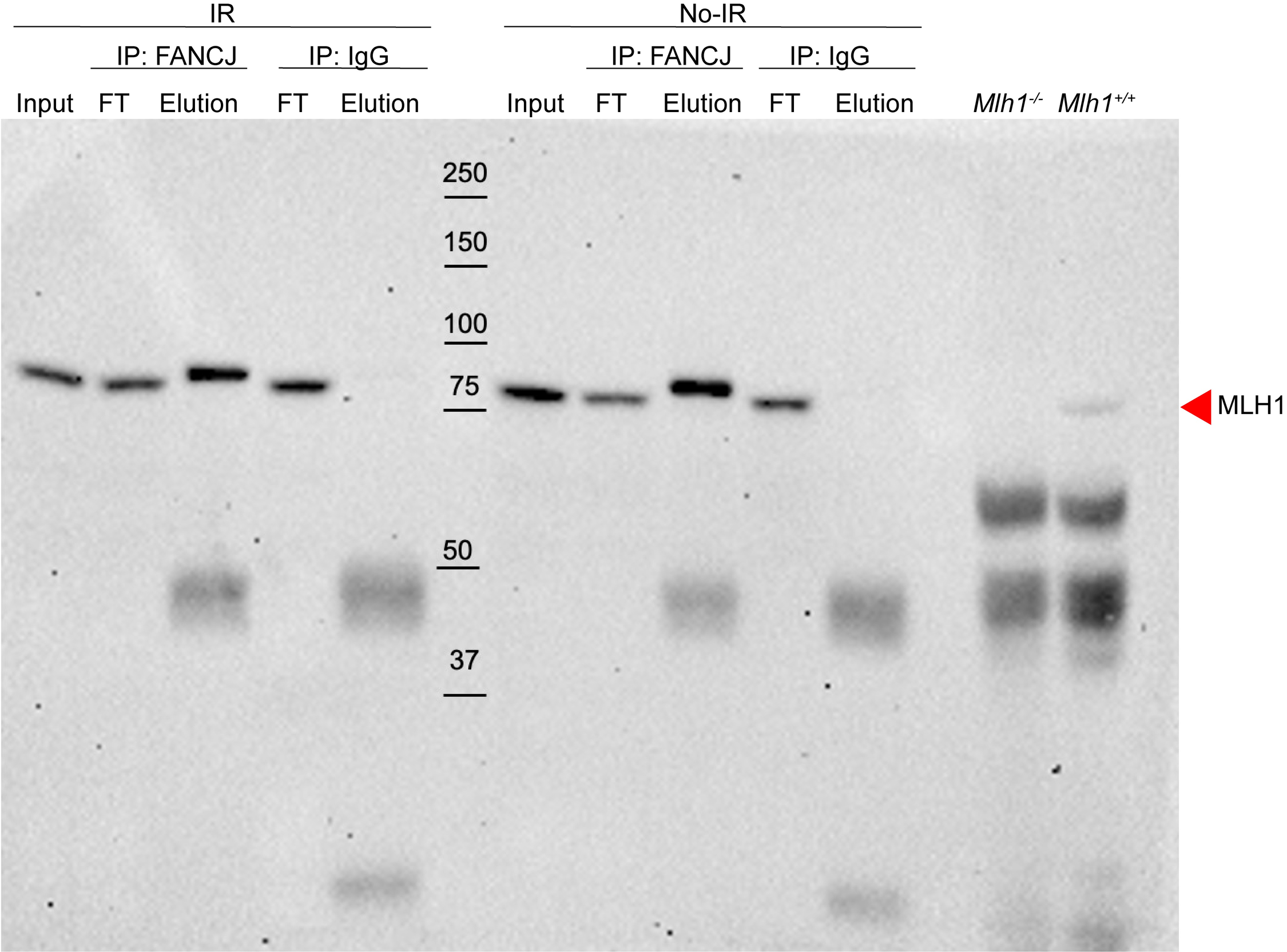

Supplement: S5 Fig — Lysate from irradiated (IR) and untreated (No-IR) pre-B cells was immunoprecipitated using antibody against FANCJ. The input lysates, flow throughs, and elutions were run on an 8% bis-acrylamide gel. The membrane was blotted with an antibody against MLH1. Red arrow points to MLH1 bands. Lysate from Mlh1+/+ and Mlh1-/- mouse whole testis lysates was also run as a positive and negative control. Two biological replicated were performed for FANCJ-IPs from pre-B cells. (TIF) [file pgen.1011175.s005.tif]

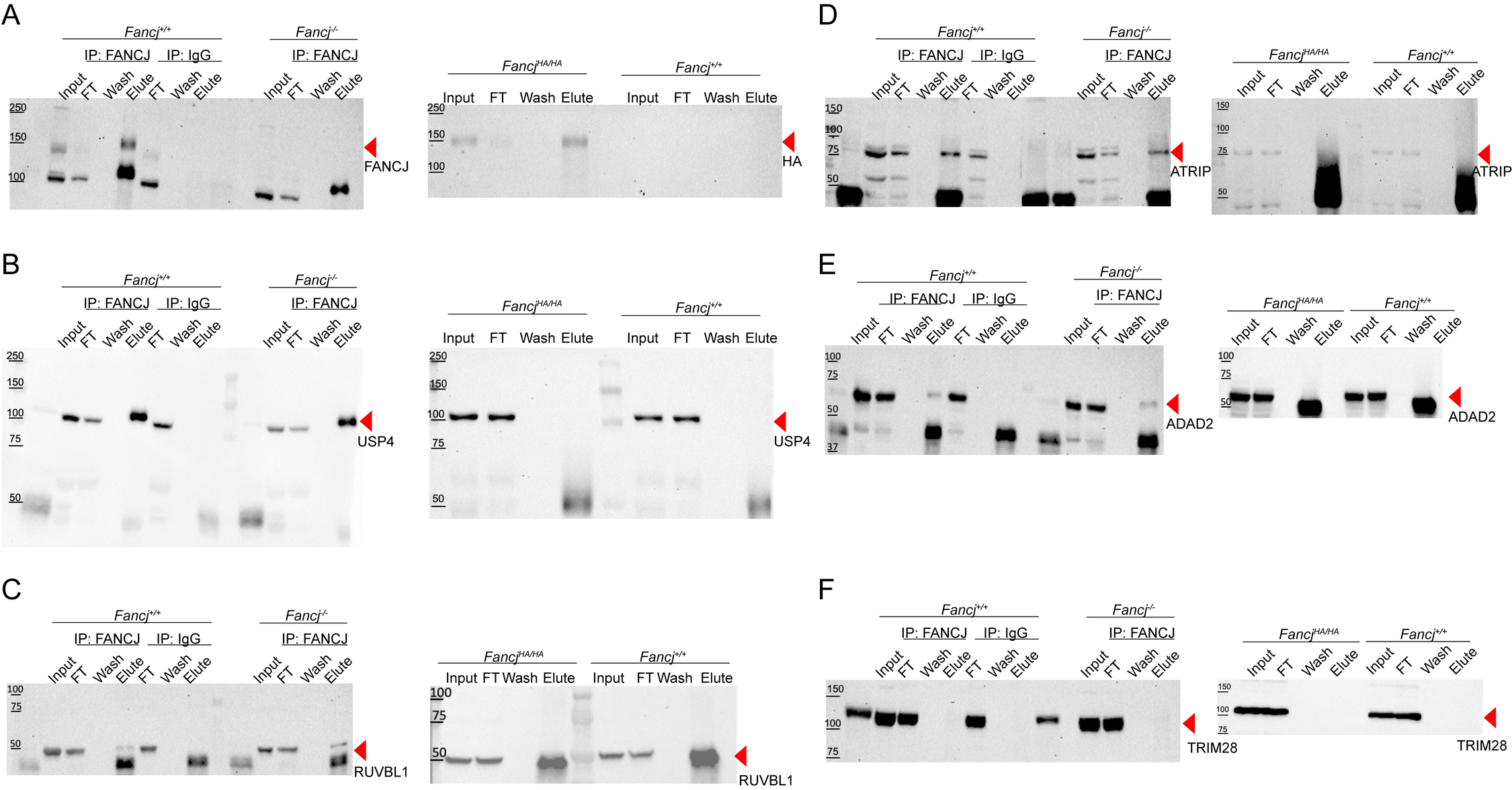

Supplement: S6 Fig — Western blots of immunoprecipitation experiments to validate initial FANCJIP-MS experiments. Each set of validations consisted of 5 separate IPs: FANCJ-IP and IgG-IP from Fancj+/+ whole testis lysate; FANCJ-IP from Fancj-/- whole testis lysate; and HA-IP from FancjHA/HA and Fancj+/+ whole testis lysates. Membranes were blotted with antibodies to: A) FANCJ and HA (to validate the IPs), B) USP4, C) RUVBL1, D) ATRIP, E) ADAD2, and F) TRIM28. (TIF) [file pgen.1011175.s006.tif]

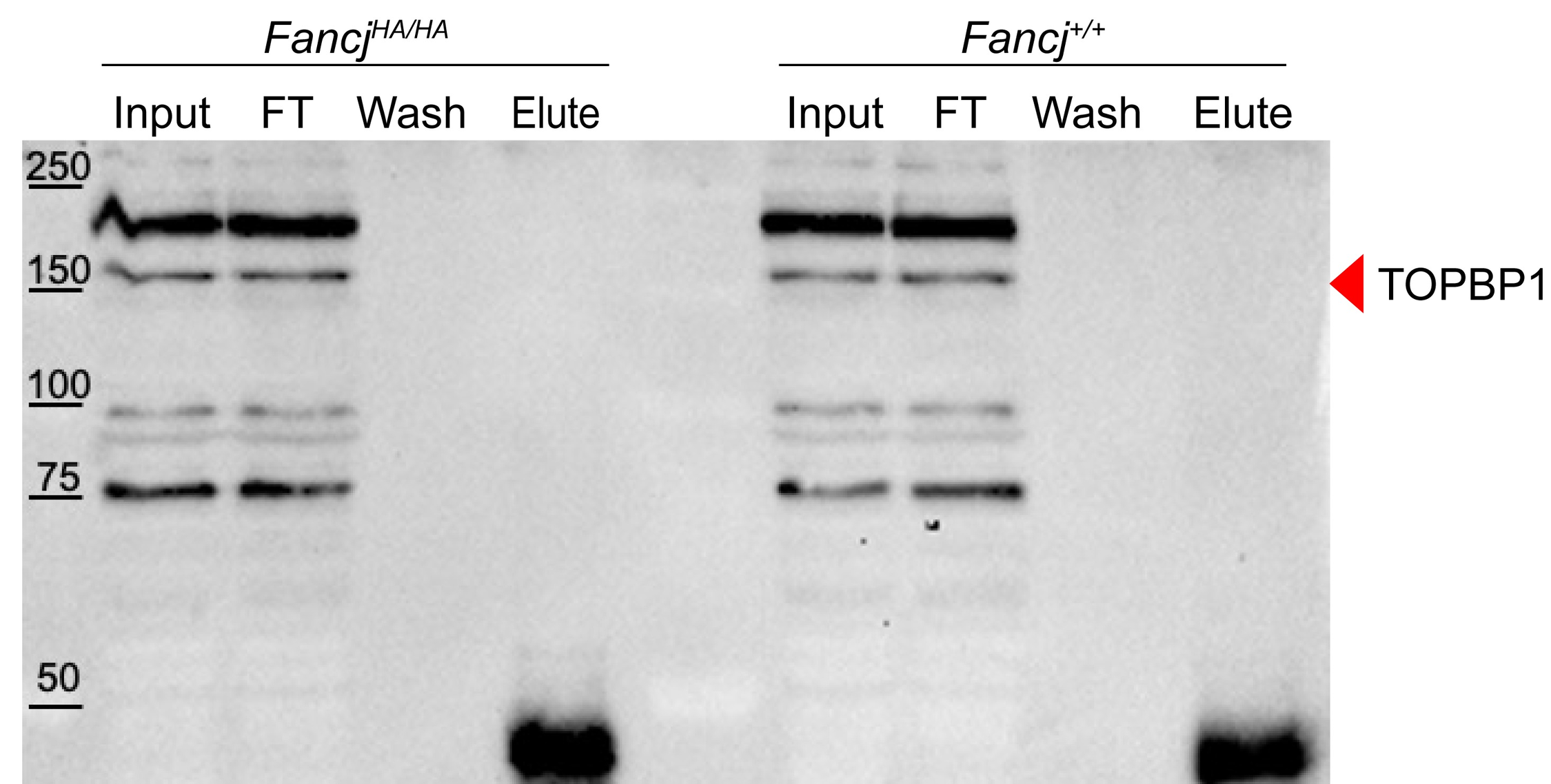

Supplement: S7 Fig — Western blot of HA-IP using whole testis lysate from FancjHA/HA and Fancj+/+mice. Membrane was blotted with antibody against TOPBP1. Red arrow points to expected TOPBP1 band size (~169 kDa). Two biological replicates of the IP and blot were performed. (TIF) [file pgen.1011175.s007.tif]
